# The Relationship of Epicardial Adipose Tissue and Cardiovascular Disease in Chronic Kidney Disease and Hemodialysis Patients

**DOI:** 10.3390/jcm11051308

**Published:** 2022-02-27

**Authors:** Kultigin Turkmen, Hakan Ozer, Mariusz Kusztal

**Affiliations:** 1 Division of Nephrology, Department of Internal Medicine, Meram Medical Faculty, Necmettin Erbakan University, Konya 42090, Turkey; hakanozer724@gmail.com; 2 Department of Nephrology and Transplantation Medicine, Wroclaw Medical University, 50-367 Wroclaw, Poland; mariusz.kusztal@umed.wroc.pl

**Keywords:** epicardial adipose tissue, cardiovascular morbidity and mortality, hemodialysis

## Abstract

Cardiovascular diseases remain the most common cause of morbidity and mortality in chronic kidney disease patients undergoing hemodialysis. Epicardial adipose tissue (EAT), visceral fat depot of the heart, was found to be associated with coronary artery disease in cardiac and non-cardiac patients. Additionally, EAT has been proposed as a novel cardiovascular risk in the general population and in end-stage renal disease patients. It has also been shown that EAT, more than other subcutaneous adipose tissue deposits, acts as a highly active organ producing several bioactive adipokines, and proinflammatory and proatherogenic cytokines. Therefore, increased visceral adiposity is associated with proinflammatory activity, impaired insulin sensitivity, increased risk of atherosclerosis, and high morbidity and mortality in hemodialysis patients. In the present review, we aimed to demonstrate the role of EAT in the pathophysiological mechanisms of increased cardiovascular morbidity and mortality in hemodialysis patients.

## 1. Epicardial Adipose Tissue

Epicardial adipose tissue is the accumulation of adipose tissue between the outer wall of the myocardium and the visceral layer of the pericardium [1]. It is derived from splanchnopleuric mesoderm and contains adipocytes, neurohumoral, stromovascular, and immune system cells [2,3]. It is frequently found near the main branches of the coronary arteries on the atrio-ventricular surfaces and in the inter-ventricular grooves [4]. EAT volume accounts for 15–20% of normal heart volume and EAT mass accounts for approximately 1% of total AT mass [5]. Age, waist circumference, ethnicity, and heart mass are independent predictors of EAT volume [5]. EAT thickness varies between 5–7 mm on the right ventricular free wall and 10–14 mm in the atrial-ventricular and inter-ventricular grooves in healthy individuals. The amount of these deposits increases in diabetic, hypertensive, and obese individuals [6].

### Imaging of Epicardial Adipose Tissue

Although it is not a method recommended by the guidelines for EAT measurement, transthoracic echocardiography is frequently the preferred method due to its ease of access, non-invasive nature, and low cost. This method has some drawbacks. The most significant limitations of EAT measurement by echocardiography are that EAT is not equal in all areas of the heart, the echocardiographer’s experience influences measurement results, the difficulty of the procedure in obese individuals, and the lack of equal densities of epicardial adipose tissue in all areas of the heart [7,8].

EAT volume was significantly correlated with intra-abdominal visceral adipose tissue deposition compared to other measurements in a study that compared volume and thickness measurements from different cross-sectional areas of EAT using a computer tomography method [9]. As a result of all of this information, EAT volume measurement stands out as the best method for determining the amount of EAT [10,11,12,13].

EAT volume was found to be more valuable in cardiovascular disease risk assessment than other EAT measurement methods in a meta-analysis of nine studies published by Nerlekar et al. [11]. CT and MR can also be used to measure EAT volume, and both methods provide a more accurate and volumetric measurement of epicardial fat tissue than transthoracic echocardiography. Although CT and MR imaging are more sensitive and specific than echocardiography, they are also more expensive and time-consuming [10]. The radiation exposure of tomography methods, in particular, and the time-consuming nature of MR imaging, limit their use.

The evaluation of EAT with echocardiography is commonly used in the general population due to its ease of access, high reproducibility, and reliability profile; however, there is insufficient data on the comparison of echocardiography and other imaging methods for the evaluation of EAT in end stage renal disease (ESRD) patients receiving hemodialysis treatment [6]. In another recent study, it was emphasized that investigating CVD risks by detecting cardiac fibrosis or steatosis may be useful in patients who do not have a known CVD risk such as diabetes or proteinuria [14].

## 2. The Relationship between Epicardial Adipose Tissue and Inflammation

**Figure 1:**
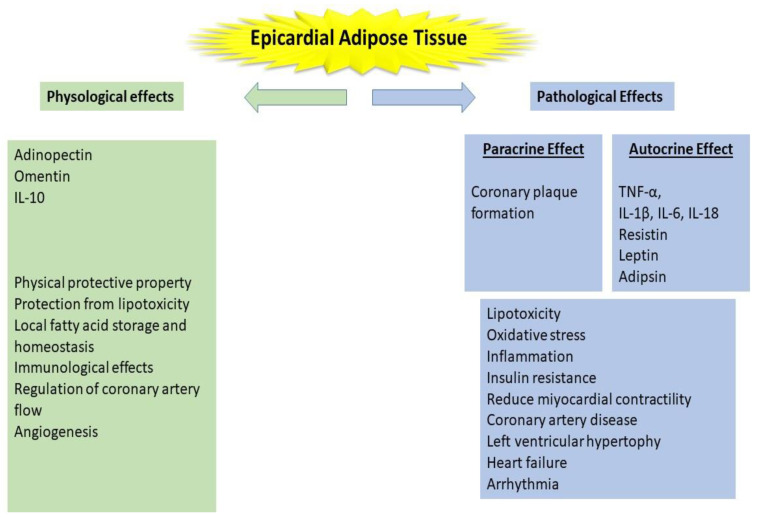
Pathophysiological features of epicardial adipose tissue in chronic kidney disease.

In healthy people, it is thought that EAT has a physical protective barrier role for the heart, has anti-inflammatory effects when the accumulation is mild, and causes pro-inflammatory and pro-atherogenic effects that cause cardio-metabolic events when the amount increases later in life [15]. In human and animal experiments, it has been determined that EAT exhibits predominantly brown adipose tissue characteristics in the early stages of life. Although white adipose tissue features dominate EAT with age, it is known that beige adipocytes, a new class of adipocyte with brown adipocyte tissue features, also exhibit their molecular and biological features in adulthood [16]. These inflammatory and anti-inflammatory events are attributed to EAT-secreted substances such as adinopectin, leptin, omentin-1, nitric oxide, and angiotensinogen, which have both autocrine and paracrine effects [17]. EAT, which is found in low amounts in healthy people, has beneficial effects on both immunological and coronary blood flow, as well as having cardioprotective properties with the use and storage of free fatty acids. Normal epicardial adipocytes secrete adiponectin in the presence of low oxidative stress (Figure 1).

Adiponectin protects cardiomyocytes from hypertrophic stimuli, as well as reduces inflammation and fibrosis in the coronary arteries and myocardium, lowering the risk of adverse clinical events. In other words, it has anti-inflammatory properties [18,19]. EAT acts as a buffering system in physiological conditions, such as removing and storing excess free fatty acids that are toxic to the myocardium, and it is involved in increased free fatty acid supply to the myocardium with increasing ischemia [20]. Adiponectin secretion decreases as biological structures change, while the synthesis of proinflammatory adipokines (leptin, tumor necrosis factor- (TNF-), interleukin 1- (IL1-), and interleukin-6 (IL1-6), and resistin) increases [21]. It is believed that EAT causes atherosclerotic areas with a local effect in the surrounding coronary arteries, as well as heart failure due to the oxidative stress it causes on the myocardium [17,22].

Proinflammatory cytokines are released from EAT rather than subcutaneous and pericardial adipose tissue in subjects with cardiovascular disease or at high risk of cardiovascular disease, according to studies in subjects with cardiovascular disease or at high risk of cardiovascular disease [3,21]. This means that the amount of EAT is more closely related to cardiovascular events than the total amount of adipose tissue in the body.

## 3. The Relationship between Epicardial Adipose Tissue and Cardiovascular Disease

While numerous studies have demonstrated a link between EAT volume and the development of cardiovascular events, it is unclear whether the role of EAT accumulation in cardiovascular disease is independent of other visceral adipose tissue deposits [11,20,23,24,25]. In a study comparing visceral adipose tissue (VAT) and EAT, diabetics had significantly larger adipocyte size in EAT than nondiabetics, and both VAT and EAT had predominantly inflammatory features in the diabetic group [26]. A meta-analysis of nine studies involving over 3500 patients revealed a link between increased EAT volume and CAD [11], as well as a link between increased EAT volume and high-risk coronary plaque formation [25]. EAT was discovered to be an independent risk factor for CVD in a meta-analysis of over 40,000 patients, and EAT volume was also linked to coronary artery calcification and myocardial ischemia [27]. According to recent research, EAT is an independent predictor of coronary events and left ventricular dysfunction, but visceral adipocyte tissue accumulation is not an independent risk factor for cardiovascular events [28,29,30,31]. On the basis of the mechanism by which EAT causes cardiovascular events, pathogenetic processes involving inflammation, insulin resistance, and oxidative stress are demonstrated [32,33,34]. With the inflammatory adipokines and cytokines it secretes, EAT causes both paracrine and autocrine effects in close proximity. EAT is not always distributed symmetrically around the entire heart. Atherosclerotic plaques are becoming more common, particularly in areas where EAT accumulation is high. This lends credence to the theory that EAT causes inflammation via paracrine effects [20,35].

Epicardial adipose tissue’s atherogenic effects are caused not only by its anatomical proximity to plaque, but also by intense proinflammatory activity. Proinflammatory adipokine production of epicardial adipose tissue is significantly higher in people with cardiovascular disease than subcutaneous adipose tissue. This suggests that epicardial adipose tissue, rather than subcutaneous fat deposition, is a better predictor of cardiovascular disease. [3,21].

Proinflammatory adipocytokines such as leptin, adipsin, TNF-, IL-1, IL-6, IL-18, and resistin are released by epicardial adipose tissue, resulting in a systemic inflammatory response. Systemic inflammation also causes a positive feedback mechanism that leads to the accumulation of epicardial adipose tissue [21,36]. Many previous studies conducted by our group concluded that the relationship between EAT and CVD frequency was caused by concurrent inflammatory events [37,38,39,40].

Oxidative stress is another mechanism by which EAT may cause atherosclerosis. It was discovered that the epicardial adipose tissue of people with CAD had higher levels of reactive oxygen radicals and lower levels of antioxidant enzymes than the subcutaneous adipose tissue of the same people [41]. With increasing oxidative stress and hypoxia, EAT’s properties change and it becomes inflammatory and proatherogenic. EAT, through the inflammatory cytokines it secretes, causes cardiometabolic events in addition to increased plaque formation in the coronary arteries it is close to [15,17,18,20]. The cardiometabolic events and increased CVD risk caused by EAT are attributed to both autocrine and paracrine effects.

Obesity is linked to an increased risk of CVD. Obese people consume more EAT than people of normal weight. It is well known that the visceral adipocyte index, a key indicator of visceral fat deposits, is linked to adipocytokines and inflammatory molecules. In this context, VAI is linked to cardiometabolic risk and is a better predictor of cardiovascular events than waist circumference or BMI alone [42]. EAT measurement can be used to estimate visceral adipocyte tissue because it has been shown to correlate with it. Visceral fat storage is also linked to epicardial fat deposition via lipid, adipokine, proinflammatory, and oxidative factors secretion [7,43,44]. CKD is a condition that increases the risk of malnutrition. Although obesity is a significant risk factor for CAD, the relationship between epicardial fat and the development of high-risk obstructive plaque is independent of obesity [45,46,47]. These findings show that epicardial fat plays a role in atherosclerotic plaque formation that is independent of visceral adipose tissue deposition.

One of the important pathways of the relationship between EAT and CVD is the renin-angiotensin system (RAS). Secretory products secreted from EAT in diabetic individuals increase cardiac fibrosis by activating the renin–angiotensin system in cardiac myocytes. RAS activation causes activation of inflammatory macrophages by mRNA remodeling and increased inflammatory state causes CVD through insulin resistance. In addition to insulin resistance, pathophysiological pathways have also been associated with lipotoxicity and oxidative stress [48]. It has been shown in a recent study that this effect can also be reduced with RAS blocker treatments [49]. In addition, the study showing that angiotensin converting enzyme hyperactivity in human EAT is associated with cardiac dysfunction supports these data [50]. Another mechanism revealed in recent years is that people with a history of CVD have a higher expression of glucagon-like peptide (GLP) in EAT [51]. Studies revealing the relationship between RAS activity and EAT CVD in CKD patients are insufficient in the literature.

Dyslipidemia is another well-known traditional risk factor for cardiovascular disease. EAT was found to be inversely correlated with HDL cholesterol and positively correlated with LDL cholesterol and TG in studies examining the relationship between cholesterol levels and CVH and EAT [27,30,44,52,53].

EAT and cardiovascular diseases are not only linked by coronary artery disease; there is also a link between heart failure, arrhythmia, and EAT [54]. EAT is thought to cause arrhythmias by compressing surrounding tissue and heart failure by causing oxidative stress on the myocardium [17,22]. EAT is linked to changes in myocardial function and may be a sign of diastolic dysfunction [55].

## 4. The Role of Epicardial Adipose Tissue in Chronic Kidney Disease Patients

The reasons and mechanisms for the increase in EAT levels in CKD patients are too complex to be attributed to a single cause, and many pathways are thought to be still unknown. When our group’s studies and other studies in the literature are considered, it is possible to conclude that the most important reason for increased EAT in CKD patients is increased inflammation in chronic kidney disease [37,38,39,40]. Furthermore, diabetes, hypertension, obesity, metabolic syndrome, and increased volume excess, which frequently accompany chronic kidney disease, are associated with an increase in EAT. It is unclear whether EAT is the result of CKD-induced metabolic disturbances or a tissue accumulation that causes these metabolic disturbances.

EAT is an endocrine organ that can cause inflammatory reactions [15,17,21]. Many studies have shown that patients with end-stage renal disease have a chronic inflammatory process, and that these inflammatory processes are associated with the amount of EAT [37,38,39,40,56,57]. In many studies conducted with our own group on this subject, we have shown that these elevated inflammatory markers are associated with EAT in both HD and peritoneal dialysis patients [37,38,39]. In this patient group, there is now a link between elevated IL-6, C-reactive protein (CRP), and ferritin levels and EAT accumulation [38]. Furthermore, inflammation caused by pro-atherogenic and bioactive molecules may be linked to increased albuminuria and worsening renal functions by causing endothelial dysfunction [53]. Colak et al. [56] discovered that inflammatory cytokines were associated with EAT in HD patients, and they also discovered that EAT thickness was higher in HD patients than in renal transplant patients with a similar follow-up period. A similar relationship between inflammatory cytokines and EAT was not found in the transplant patient group of the study. This finding supports the notion that increased inflammatory load in dialysis patients causes EAT accumulation. The fact that EAT thickness in renal transplant patients was found to be similar to the control group suggests that the proinflammatory state of hemodialysis has an inducing effect on EAT other than uremia. Different studies looked into the effects of uremia on EAT by intensifying HD treatment. At the end of 12 months, there was no significant change in EAT amounts in patients who were switched from 4 h of intermittent dialysis 3 days a week to 8 h of nighttime HD. This study is significant because it is the first to examine whether increasing the intensity of HD treatment causes a change in the amount of EAT [58]. The lack of significant changes in inflammation markers at the end of another study with a similar design could imply that the role of inflammatory pathways, rather than uremia, is more important in increased EAT accumulation in HD patients [57].

Obese people consume more calories. Despite increased malnutrition, EAT volume is higher in CKD patients than in the healthy population. In this context, it can be concluded that body weight is not the only or the most important determinant of EAT volume [30,56]. Decreased food intake in CKD patients, together with increased catabolic processes secondary to inflammation and a decrease in muscle mass, increases the negative effects of metabolic disorders and explains the increased EAT accumulations. In patients with CKD, visceral adipose tissue distribution and accumulation change over time with the effect of uremia. Visceral adipose tissue deposition is inversely proportional to e-GFR [59]. A recent meta-analysis discovered that the EAT volume was higher in stage 4–5 patients than in stage 3 patients [60]. The study in which our group demonstrated the relationship between EAT and malnutrition, inflammation, atherosclerosis, and vascular calcification in ESRD patients is the first in the literature to demonstrate that EAT accumulation and CVD risks increase in this patient group despite a low BMI [61]. In CKD patients, decreased food intake combined with an increased catabolic process leads to protein and muscle loss over time, eventually leading to insulin resistance. Along with insulin resistance, it is believed that changes in fatty acid metabolism and inflammatory pathways may lead to an increase in EAT [62].

Diabetes and hypertension are the most common causes of chronic kidney disease. Diabetes and hypertension patients consume more EAT than the general population [6]. The fact that the amount of EAT in diabetic CKD patients is higher than in non-diabetic CKD patients, according to a study on the subject, indicates that diabetes is an important factor that increases the amount of EAT in CKD patients [63]. The majority of the studies evaluating EAT in ESRD had diabetic and/or hypertensive patients as participants. This begs the question of whether there is an increase in EAT as a result of CKD, diabetes, or hypertension. Mazurek et al. [3] demonstrated that the relationship between EAT and CAD is independent of diabetic status, lending credence to the notion that EAT increases in CKD patients for reasons other than diabetes

In a study examining the change in EAT accumulation in dialysis patients with exercise that lowers the risk of CVD, it was discovered that EAT accumulations decreased in the follow-up in patients who exercised on days when they did not receive HD treatment. EAT thickness was thought to decrease with decreasing oxidative stress as a possible mechanism [52].

Fluid overload is a serious condition that increases the risk of CVD in dialysis patients, particularly by causing LVH. EAT and hypervolemia were found to be correlated in the only study that looked at the relationship between hypervolemia and EAT [56]. It is possible that increasing hypervolemia causes LVH and creates an inducing force on EAT accumulation due to increased myocyte energy demand.

Numerous dialysis-specific studies have found that EAT accumulation is higher in HD patients than in the general population or in kidney transplant patients [39,56,63,64]. Furthermore, a link was discovered between the length of stay in HD and EAT thickness [65]. In different studies, the effects of dialysis treatment durations and doses on the amount of EAT produced contradictory results [58,66]. This difference could be due to the presence of different diseases affecting the amount of EAT in the patient groups, as well as differences in the patients’ dialysis treatment history.

## 5. Relationship between CVD and EAT in ESRD Patients and Patients Receiving Hemodialysis Treatment

Diabetes and hypertension are the most common causes of chronic renal failure, and the presence of these diseases, in addition to kidney failure, poses an additional cardiovascular risk. Cardiovascular diseases account for nearly half of all deaths in CKD patients, and a history of CKD increases the risk of cardiovascular disease by 1.5 to 3.5 times [67,68]. Dialysis patients have a higher rate of coronary artery disease, congestive heart failure, sudden death, and arrhythmias than the general population [69].

**Figure 2:**
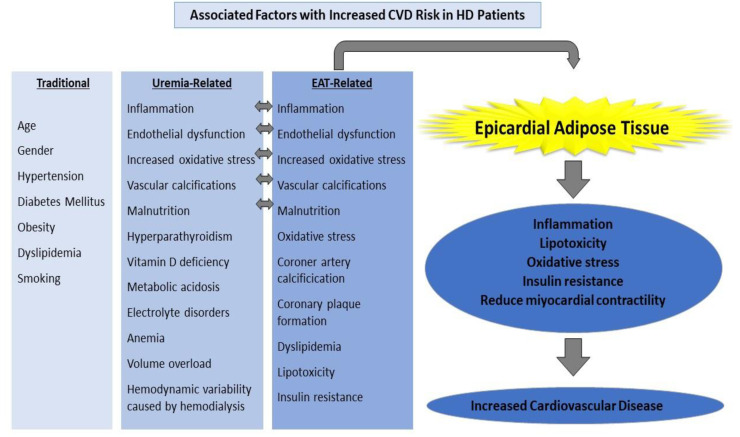
Factors associated with an increased CVD risk in HD patients.

Traditional risk factors such as advanced age, smoking, obesity, dyslipidemia, and family history are insufficient to explain why CKD patients have an increased risk of CVD. Common metabolic disorders in CKD patients include oxidative stress, nonspecific chronic inflammation, uremic toxins, anemia, malnutrition, insulin resistance, metabolic acidosis, hyperparathyroidism, hyperhomocystinemia, and vitamin D deficiency [68,70]. Atherosclerosis, endothelial dysfunction, hypertension, diabetes mellitus (DM), and micro-macrovascular complications caused by diabetes, chronic inflammation, coronary artery calcification (CAC), and left ventricular hypertrophy (LVH) are the most important risk factors for CVD in patients with chronic kidney disease [71,72]. Atherosclerosis, inflammation, and vascular calcification are the most common risk factors in the pathogenesis of CVD in ESRD patients [71] (Figure 2).

End-stage renal disease is distinguished by a persistent and non-specific inflammatory process. Many inflammatory cytokines, especially TNF-α, IL-1-β, and IL-6 increase in CKD [73,74]. Inflammatory cytokines are linked to endothelial dysfunction, atherosclerosis, and, eventually, an increase in cardiovascular disease. Hemodialysis, a renal replacement therapy used in end-stage renal disease, is associated with additional risks such as increased oxidative stress and endothelial dysfunction, as well as cardiovascular risks caused by kidney failure. Hemodynamic instability during dialysis is another significant event that raises cardiovascular risk [75].

Dialysis patients have a higher rate of coronary artery disease, congestive heart failure, sudden death, and arrhythmias than the general population [69]. Traditional risk factors such as age, gender, hypertension, obesity, dyslipidemia, or smoking alone cannot explain the increased frequency of CVD in dialysis patients. Inflammation, endothelial dysfunction, increased oxidative stress, and vascular calcifications are cited as major causes of these elevated CVD risks. Malnutrition, hyperparathyroidism, vitamin D deficiency, long-term and severe uremia, metabolic acidosis, electrolyte disorders, anemia, long-term volume overload, and hemodynamic variability caused by hemodialysis all contribute to an increase in the incidence of cardiovascular disease [68,70,71,72]. Chronic kidney disease itself is also considered a CVD risk equivalent.

In a study of diabetic kidney disease patients conducted by Sasso et al. [76], the incidence of fatal/non-fatal CVD in patients who received multifactorial intensive therapy against cardiovascular disease risk factors was 53% lower than in the group that received standard treatment. In this study, intensified and standard treatments were compared against known standard and manageable risk factors for CVD. This study compared intensified and standard treatments to known standard and manageable risk factors for CVD. [77]. However, we know that dialysis patients consume more EAT than the general population. EAT was linked to many known risk factors in this patient group, including age, low HDL cholesterol, high LDL cholesterol and triglyceride levels, smoking, and BMI [30,44,52,53]. The increased EAT volume in dialysis patients can be viewed as both a result and a cause of these pathways, which increase the frequency of CVD. In HD patients, the relationship between EAT and CVD is primarily focused on epicardial adipose tissue enlargement, inflammation, and hypermetabolic activity, which is led by systemic inflammation [78].

In a recent study, 221 ESRD patients were tested to see if EAT radiodensity and volume could predict long-term mortality. In patients with ESRD, EAT radiodensity was an independent predictor of all-cause mortality. EAT volume, on the other hand, was not linked to mortality [79]. This study is useful in terms of linking EAT to long-term mortality. Similarly, in another study examining the relationship of EAT thickness with mortality in ESRD patients over a 10-year period, patients with an EAT thickness greater than 11.45 mm lived approximately 2 years longer than those with an EAT thickness less than 11.45 mm [80].

In the general population, there is a positive relationship between age and the amount of EAT consumed [81]. When a similar relationship was investigated in HD patients, it was discovered that the amount of EAT was also related to increasing age [82]. There is a redistribution of visceral fat stores with aging, which is thought to increase the amount of EAT.

A 4-year follow-up period was included in the first study to describe the relationship between EAT and mortality in hemodialysis patients. EAT volume has been shown to be an independent predictor of mortality in hemodialysis patients. The study found that every 10cc increase in EAT volume resulted in a 6% increase in the risk of death. Researchers believe that this increased risk is due to EAT’s effects on vasculopathy and CAC [30]. CAC is a type of long-term vascular calcification that can be detected early in ESRD patients and is thought to contribute to both increased CVD and mortality. We previously demonstrated a link between EAT and coronary artery calcification in patients with end-stage renal disease receiving hemodialysis and peritoneal dialysis in three studies [39,61,64]. EAT was found to be associated with vascular calcification in HD patients in another study with a similar design, but it could not be determined as a predictor of mortality [83]. The smaller number of patients in this study reduces the study’s data reliability.

The most common risk factors in the pathogenesis of CVD in dialysis patients are atherosclerosis, inflammation, and vascular calcification [71]. Coronary flow reserve is regarded as a marker of endothelial dysfunction. EAT thickness in hemodialysis patients was found to be inversely proportional to CFR in a study examining the relationship between EAT and coronary flow reserve [84]. Coronary flow reserve is regarded as a marker of endothelial dysfunction. EAT thickness in hemodialysis patients was found to be inversely proportional to CFR in a study examining the relationship between EAT and coronary flow reserve [15,17,18,20]. EAT is not usually found symmetrically around the heart. Atherosclerotic plaques are becoming more common, particularly in areas where EAT accumulation is high [20]. This raises the possibility that EAT is causing inflammation through a paracrine effect [35]. This hypothesis is supported by the observation that EAT thickness is significantly associated with the presence and severity of CAD, as well as by the ability to stop CAD progression following epicardium resection [85].

The amount of EAT increases as the stage of renal failure progresses. It is well known that dialysis patients consume more EAT than the general population. Saritas et al. [27] discovered that eGFR was independently and inversely related to EAT volume, and they hypothesized that EAT could be a marker of the uremia-specific component of CV risk based on this finding.

In the comparison of hemodialysis patients and transplant patients at the same follow-up period, EAT levels were found to be higher in dialysis patients and associated with inflammatory variables; EAT levels in transplant patients were found to be both unrelated to inflammatory markers and similar to the healthy population. This clearly demonstrates the role of increased inflammatory processes in dialysis patients in the EAT and CVD equation. [56]

Obese people consume more calories. Obesity is a risk factor for CVD but not an independent predictor of EAT [6]. Dialysis patients are at risk of malnutrition due to reduced food intake and increased inflammation. Weight loss due to malnutrition and dialysis is linked to an increased risk of death. Muscle wasting, which occurs as catabolic processes increase, leads to an increase in insulin resistance [62]. Simultaneously, the combination of increased uremia and decreased GFR alters the distribution of visceral adipose tissue [59]. Our group’s two studies clearly demonstrated the link between malnutrition and EAT in HD patients. In the first study, our group discovered a link between low albumin levels and increased EAT accumulation in HD patients; in the second study, we discovered a link between EAT and malnutrition, elevated inflammatory markers, atherosclerosis, and vascular calcification in HD patients [61,86]. Despite having a low BMI, HD patients are at an increased risk of EAT accumulation and CVD [61]. Insulin resistance caused by malnutrition in dialysis patients increases metabolic events and CVD risks. While visceral adipocyte tissue accumulation is not an independent risk factor for cardiovascular events, there are numerous studies showing that EAT is an independent predictor of cardiovascular events [28,29,30,31]. Furthermore, EAT produces more inflammatory cytokines than subcutaneous adipose tissue [21]. Based on these findings, EAT accumulation in dialysis patients is more valuable than other visceral adipose tissue deposits in predicting increased CVD frequency, and it is independent of body weight.

Comorbidity diseases such as diabetes and hypertension that accompany this patient group are one of the most important reasons for the high CVD risks in dialysis patients. Diabetes and hypertension patients consume more EAT than the general population. The amount of EAT is higher in diabetic and hypertensive individuals than in the healthy population [6]. Different results have been obtained in studies conducted on whether diabetes affects the amount of EAT in renal failure. Various studies on whether diabetes affects the amount of EAT in renal failure have yielded conflicting results. While Tonbul et al. [63] discovered that EAT was higher in diabetic kidney patients than in non-diabetic patients, Mazurek et al. [3] demonstrated that the relationship between EAT and CAD in CKD is independent of diabetic status and suggested that there are other factors other than diabetes that influence the amount of EAT in CKD patients. While Tonbul et al. found EAT higher in diabetic kidney patients than in non-diabetic patients, Mazurek et al. showed that the relationship between EAT and CAD in CKD is independent of diabetic status and suggested that there are additional reasons independent of diabetes in determining the amount of EAT in CKD patients. There is substantial evidence in the literature that systolic blood pressure and LVH are related to EAT thickness [87]. Hypertension (HT) is common in patients with end-stage renal disease (ESRD). The increase in EAT mass to meet the increased myocardial energy requirement after the increase in left ventricular wall thickness caused by high blood pressure is one of the most important mechanisms that can explain the relationship between EAT and LVH [88].

Dyslipidemia is a metabolic disorder that is commonly found in dialysis patients and is a known risk factor for CVD. EAT in dialysis patients was found to be inversely correlated with HDL cholesterol and positively correlated with LDL cholesterol and TG in studies examining the amount of EAT and cholesterol levels [27,30,44,52,53]. The link between hyperlipidemia and EAT is well established, and the situation in dialysis patients is similar to that of the general population.

Exercise lowers the risk of CVD as well as the amount of EAT. The only study that looked at the relationship between exercise and the amount of EAT in dialysis patients discovered that standard exercise on days when HD treatment was not given reduced the amount of EAT [52]. The authors interpreted the possible cause of this situation as decreased EAT thickness due to decreased oxidative stress.

Between dialysis sessions, dialysis patients with insufficient renal residual function experience an average of 3 kg of hypervolemia. LVH develops over time as a result of the chronic hypervolemia state. EAT and hypervolemia were found to be correlated in the only study that looked at the relationship between hypervolemia and EAT [56]. It is possible that increased hypervolemia causes LVH and that this condition creates an inducing force on EAT accumulation due to myocytes’ increased energy requirement.

Not only is there a link between EAT and coronary artery disease, but there is also a link between heart failure and arrhythmia and EAT, according to research. There was a significant relationship discovered between the amount of EAT and atrial fibrillation [54]. EAT is thought to cause arrhythmias due to the pressure it exerts on surrounding tissue, and it is also thought to cause heart failure due to the oxidative stress it causes on the myocardium [17,22].

The accumulation of EAT increases in direct proportion to the duration of HD treatment [65]. The findings of studies looking into the effects of reducing uremia by increasing hemodialysis treatment on EAT are contradictory [58,66]. This difference may be due to the presence of different diseases affecting the amount of EAT in the patient groups, as well as differences in the patients’ dialysis treatment history, and it supports the idea that reducing uremia alone cannot reduce CVD risks without reducing existing inflammation [57].

Corrective lifestyle changes and medical treatments for known CVD risk factors reduce EAT as well. Severe dietary restrictions cause a greater reduction in EAT in obese individuals than BMI and waist circumference [89]. Treatment modalities that lower lipid and glucose levels also cause a decrease in the amount of EAT [90]. Statin therapy was found to have a greater anti-inflammatory effect on EAT than subcutaneous adipose tissue [91].

## 6. Conclusions

EAT is a metabolically active tissue that increases in dialysis patients and is thought to play a role in the pathogenesis of CVD. It can also be used to predict the risk of cardiovascular disease. Many factors contribute to the amount of EAT and the frequency of CVD, the majority of which are biomarkers that are difficult to measure or monitor in clinical practice. As a result, monitoring EAT will indirectly allow monitoring of these biomarkers, which have a direct effect on the development of cardiovascular events. Cardiovascular events are the leading cause of death in dialysis patients. We believe that, in this patient population, EAT follow-up, in addition to traditional CVD risk factors, may be useful in determining the risk of cardiovascular disease.

## Data Availability

Not applicable.
